# Genetic dissection of QTLs for starch content in four maize DH populations

**DOI:** 10.3389/fpls.2022.950664

**Published:** 2022-10-06

**Authors:** Xiaolei Zhang, Min Wang, Changzheng Zhang, Changjun Dai, Haitao Guan, Ruiying Zhang

**Affiliations:** ^1^Quality and Safety Institute of Agricultural Products, Heilongjiang Academy of Agricultural Sciences, Harbin, China; ^2^Institute of Advanced Agricultural Technology, Qilu Normal University, Jinan, China; ^3^Maize Yufeng Biotechnology LLC, Beijing, China

**Keywords:** maize, DH, kernel, starch, QTL

## Abstract

Starch is the principal carbohydrate source in maize kernels. Understanding the genetic basis of starch content (SC) benefits greatly in improving maize yield and optimizing end-use quality. Here, four double haploid (DH) populations were generated and were used to identify quantitative trait loci (QTLs) associated with SC. The phenotype of SC exhibited continuous and approximate normal distribution in each population. A total of 13 QTLs for SC in maize kernels was detected in a range of 3.65–16.18% of phenotypic variation explained (PVE). Among those, only some partly overlapped with QTLs previously known to be related to SC. Meanwhile, 12 genes involved in starch synthesis and metabolism located within QTLs were identified in this study. These QTLs will lay the foundation to explore candidate genes regulating SC in maize kernel and facilitate the application of molecular marker-assisted selection for a breeding program to cultivate maize varieties with a deal of grain quality.

## Introduction

Maize (*Zea mays* L.) is one of the important crops in the world. Starch is the main component of maize kernel, which accounts for about 70% of kernel weight and is the main energy source to supply adequate food to humans and feed animals and is a source of demand in bio-ethanol production and other industrial applications (Nelson and Pan, 1995; Balter, 2007). Therefore, understanding processes related to starch biosynthesis in maize kernel is of fundamental biological interest, and such knowledge also benefits agricultural applications by providing means to improve grain yield and quality in seed production (Comparot-Moss and Denyer, 2009; Jeon et al., 2010).

To elucidate genetic variation in starch biosynthesis and regulation, over the past few years, numerous quantitative trait locus (QTL) studies have been carried out using different mapping methods and populations, and many QTLs associated with starch content (SC) in maize kernel have been revealed (Liu et al., 2008; Wassom et al., 2008; Zhang et al., 2008; Li et al., 2009; Wang Y. Z. et al., 2010; Cook et al., 2012; Guo et al., 2013; Nancy et al., 2014; Yang et al., 2014; Dong et al., 2015; Alves et al., 2019). For instance, six QTLs were identified in a recombinant inbred line (RIL) population, and four genes were considered candidate genes likely acting as direct regulators of starch biosynthesis (Wang et al., 2015). A genome-wide association study (GWAS) revealed 27 QTLs involving 39 candidate genes that are associated with amylose content, including transcription factors, glycosyltransferases, glycosidases, and hydrolases (Li et al., 2018). Eight QTLs were revealed in an intermated B73×Mo17 population, and the gene encoding GLABRA2 expression modulator was nominated as the candidate gene of a major QTL by GWAS and the gene co-expression network analysis (Lin et al., 2019). Recently, in a total of 50, 18 novel QTLs were identified by integrating single linkage mapping, joint linkage mapping, and GWAS in a multi-parent population containing six RIL populations (Hu et al., 2021). Thus, although common QTLs associated with SC has been reported in different studies, unique QTLs were always specialized in the context of distinct populations and parents, which prompt us to conduct further investigations using relevant germplasm to extend our knowledge on the genetic basis of SC.

Distinct strategies of mapping populations were featured with strengths and limitations, which caused great impacts on the outputs of QTL (Odell et al., 2022). In particular, different types of populations tend to vary with two main characteristics: (1) their ability to capture genetic diversity and (2) their power to detect QTL of small effect (Odell et al., 2022). Double haploid (DH) segregating populations have been commonly used in QTL analysis for several particular advantages (Chaikam et al., 2019). Complete homozygosity of DH lines allows more accurate phenotyping over multiple locations and years compared to families in early selfing generations (Foiada et al., 2015; Yan et al., 2017). In addition, DH populations enable the removal of any residual heterozygosity, ensuring replicates are genetically identical (Odell et al., 2022). Moreover, the relatively high genetic variance in DH lines increases selection response by stabilizing the heritability of various traits during perse and test cross evaluation (Bordes et al., 2006; Gallais and Bordes, 2007; Mayor and Bernardo, 2009). In this study, we utilized four DH populations derived from the practical breeding program to further dissect the genetic basis and QTLs controlling the phenotypic variation of SC in maize kernels. We intended to mine novel alleles and genes from DH populations to improve the starch content of advanced maize germplasms in-house.

## Materials and methods

### Plant materials

Four DH populations (SC1, SC2, SC3, and SC4) including 345, 275, 134, and 258 lines, respectively, were developed from biparental crosses of eight inbred lines exhibiting the variation in SC (Table 1). These parental lines belonged to elite inbred lines that were widely used by the breeding program at Maize Yufeng Biotechnology LLC to optimize grain nutritional quality. Parents of SC1 and SC2 belonged to Reid Yellow Dent germplasm, and parents of SC3 and SC4 belonged to maize Lancaste germplasm (Table 1). Plants were grown in Liaoning province, China (LN, 40°‘82′N, 123°56′E) with three replication blocks in 2021. Each line was grown in a single-row plot with a row length of 150 and 60 cm between rows under natural field conditions. All plants in each row were self-pollinated and harvested after maturity. Kernels from the middle part of three well-grown ears were bulked for the measurement of starch. We declared that all the collections of plant and seed specimens related to this study were performed in accordance with the relevant guidelines and regulations by the Ministry of Agriculture (MOA) of the People's Republic of China.

**Table 1:** Phenotypic performance, variance, and broad-sense heritability of starch content (SC) in the four double-haploid (DH) populations.

**Trait ^a^**	**Populations**							
	**SC1**		**SC2**		**SC3**		**SC4**	
**Parents**								
means ± SD (%)	AJ517001	72.10 ± 0.47	AJ517003	75.10 ± 0.41	KB519001	73.91 ± 0.99	KB519003	72.99 ± 0.46
	AJ517002	73.67 ± 0.35	AJ517004	70.45 ± 0.51	KB519002	75.80 ± 0.34	KB519004	71.90 ± 0.89
*p*-value^*b*^	0.058^ns^		0.009^**^		0.0194^*^		0.1312^ns^	
**DHs**								
Size	345		275		134		258	
means ± SD (%)	71.18 ± 1.31		71.55 ± 2.34		73.85 ± 1.65		72.90 ± 1.98	
Range (%)	66.38–74.76		61.22–75.46		67.40–77.71		64.87–76.97	
$\sigma_{g}^{\text{2 c}}$	1.45		4.41		2.11		3.25	
$\sigma_{e}^{\text{2 d}}$	1.09		1.35		1.29		1.31	
$\sigma_{\varepsilon }^{2\text{e}}$	1.53		3.68		1.34		3.26	
*h^2^* (%)^f^	73.90		78.20		82.52		74.94	

a SC;

b P value based on a t-test evaluating two parental lines;

c genetic variance;

d environmental variance;

e residual variance;

f broad-sense heritability (h^2^);

* p ≤ 0.05,

** p ≤ 0.01, ns no significant different.

### Starch content measurement and phenotypic data analysis

The starch content in maize kernels was determined by using a near infrared reflectance (NIR) spectrometer (DA 7250, Perten Instruments Inc., Sweden). The reflectance spectra were collected in a range of 400 to 2500 nm with 10-nm intervals in the NIR region. Each sample was bulked with at least 50 kernels and scanned three times.

R Version 4.0.1 (www.R-project.org) was used to perform all statistical analyses as previously described (Zhang et al., 2021). The variances of SC were estimated by using the R ‘AOV' function. The model for the variance analysis was as follows: y = μ + α_g_ + β_e_ + ε, where α_g_ is the effect of the g^th^ line, β_e_ is the effect of the e^th^ environment, and ε is the error. The effects in the model were defined by random. The broad-sense heritability as *h*^2^ = $\sigma_{g}^{2}$*/*($\sigma_{g}^{2}$ + $\sigma_{\varepsilon }^{2}$/*e*) (Knapp et al., 1985) was calculated by using these variance components, where $\sigma_{g}^{2}$ is the genetic variance, $\sigma_{\varepsilon }^{2}$ is the residual error, and *e* is the number of environments. The best linear unbiased predictor (BLUP) value of each line was calculated to eliminate the influence of environmental effects by using a linear mixed model. The model was y_ij_ = μ + e_i_ + f_j_ + ε_ij_, where y_ij_ is the phenotypic value of individual j in environment i, μ is the grand mean, e_i_ is the effect of different environments, f_j_ is the genetic effect, and ε_ij_ is the random error. Both genotype and environment were considered as random effects in the R function “LME4”.

### Genotyping and constructing a genetic linkage map

All lines were genotyped with the GenoBaits Maize 1K marker panel containing 4,589 SNP markers that were developed by Mol Breeding Biotechnology Co., Ltd., Shijiazhuang, China (http://www.molbreeding.com/), based on genotyping by target sequencing platform in maize (Guo et al., 2019). The SNP positions in the B73 reference genome Version 3 were converted to those in Version 4 using CrossMap Version 2.0.5 (Zhao et al., 2014). SNPs with minor allele frequency (MAF) < 0.1 or missing rate > 0.6 were filtered out in each population. Finally, high-quality SNPs in each population were then used to construct the genetic linkage maps *via* the R/qtl package functions est.rf and est.map (Broman et al., 2003) with the kosambi mapping method.

### QTL mapping

The quantitative trait loci were analyzed by the composite interval mapping (CIM) method implemented in Windows QTL Cartographer 2.5 (Wang S. et al., 2010). The genome was scanned at every 1.0 cM interval between markers using a 10 cM window size. The forward and backward stepwise regressions with five controlling markers were conducted to control the background from flanking markers. The empirical logarithm of the odds (LOD) threshold was determined by the 1,000 permutations at a significance (*p* < 0.05) and used to identify the significant QTLs (Churchill and Doerge, 1994). These threshold LOD values ranged from 2.86 to 3.09 in four DH populations, respectively. For clarity, we used 3.00 as the LOD threshold for the four DH populations. With the 1.5-LOD support interval method, the confidence interval for each QTL position was estimated (Lander and Botstein, 1989). The additive × additive epistatic interactions were performed by the “ICIM-EPI” method in IciMapping Version 4.2 (Li et al., 2008).

## Results

### Phenotypic variation and heritability in kernel SC

**Figure 1:**
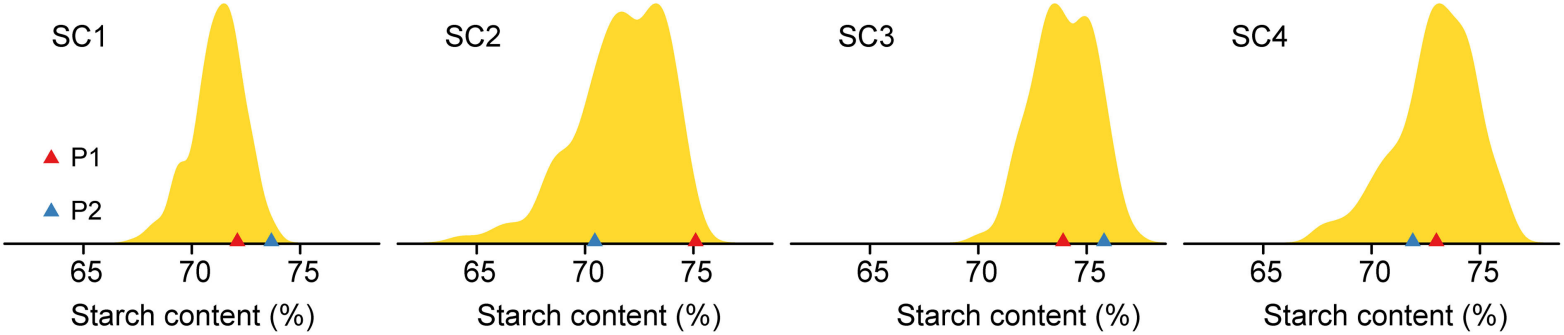
Phenotypic variations in starch content (SC) in the four double-haploid (DH) populations. The x-axis shows the SC and the color triangle represents the parents.

Eight inbred lines (SC with a range of 70.45–75.80%) were used to develop four DH populations, which included 134–345 lines, respectively (Table 1). The SC exhibited continuous and approximate normal distribution in each DH population with a range of 61.22–77.71% (Figure 1, Table 1). The analysis of variance (ANOVA) revealed that the genotype variance was greater than the environmental variance in all populations, indicating that phenotypic variations were mainly controlled by genetic factors. Broad-sense heritability estimates were calculated and showed moderate to high heritability for SC in the four DH populations, with a range of 73.90–82.52% (Table 1). Together, these results indicated that the majority of SC variations are controlled by genetic factors and are suitable for further QTL mapping.

### Genotyping and genetic linkage map

All DH lines in four populations were genotyped using the GenoBaits Maize 1K marker panel containing 4,589 SNP markers and further refined by eliminating SNPs with MAF < 0.1 or missing rate > 0.6. This resulted in a total of 776, 1,164, 831, and 1,216 polymorphic SNPs, with their precise physical positions mapped on the B73 reference sequence Version 4. In each DH population, the missing rate of SNPs in most lines was <2% (Supplementary Figure S1). Four linkage maps spanned a total of 775.88, 858.86, 627.75, and 773.21 cM genetic distances, respectively (Supplementary Figure S2). The average genetic distance between every two adjacent markers was 1.01, 0.74, 0.76, and 0.64 cM in each DH population, respectively (Supplementary Table S1).

### Identification of QTLs for SC in four DH populations

**Figure 2:**
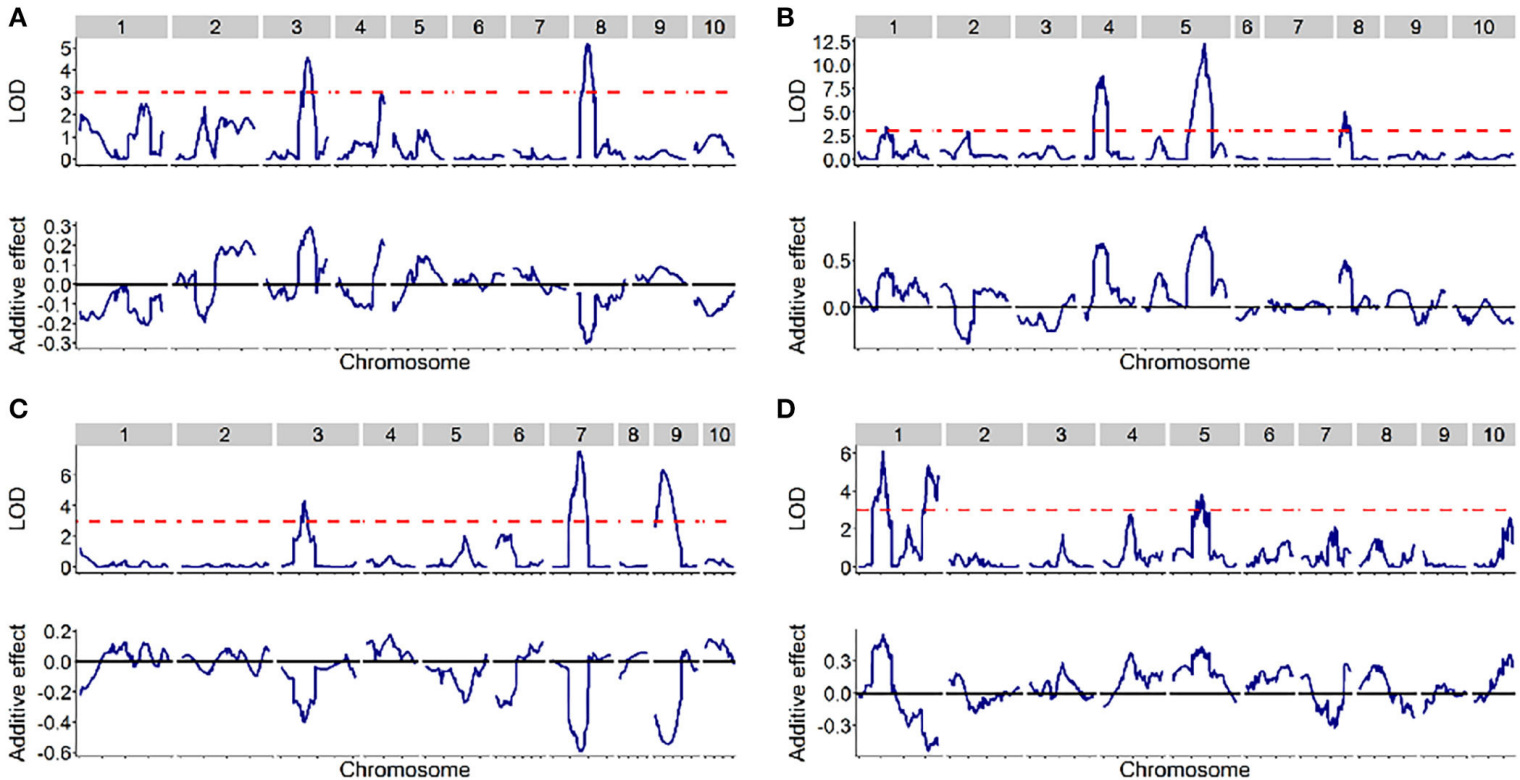
The distribution of SC quantitative trait loci (QTLs) across the entire genome in the four DH populations. The upper of each picture displays a logarithm of the odds (LOD) score (y-axis) against the physical position (x-axis) of markers, while the bottom of the picture displays the additive effect (y-axis) against the physical position (x-axis) of markers. **(A–D)** designated SC1, SC2, SC3, and SC4, respectively.

The quantitative trait locus mapping for SC in the four DH populations and the related genetic features are summarized in Table 2. In total, 13 QTLs were identified with a LOD threshold of above 3.00 in the four populations (Table 2, Figure 2) and distributed on chromosomes 1, 3, 4, 5, 7, 8, and 9. The average of QTL genetic intervals was 9.82 cM in a range of 4.95–17.20 cM. The average of QTL physical intervals was 46.51 Mb in a range of 5.60–149.78 Mb. The average of the total PVE explained by all identified QTLs in a population was 26.13 and ranged from 12.80 (SC1) to 41.57% (SC3). This was less than broad-sense heritability (Tables 1, 2), suggesting that only part of the QTLs have been detected in bi-parent populations.

**Table 2:** Individual quantitative trait locus (QTL) for SC in the four DH populations.

**Populations**	**QTL**	**Chr.^a^**	**G-Peak (cM)^b^**	**P-Peak (Mb)_V4^c^**	**G-Range (cM)^d^**	**P-Range (Mb)_V4^e^**	**LOD**	**PVE%^f^**	**Add.^g^**	**Parent ^h^+**	**PVE(%) -ALL^i^**
SC1	*qSC-1-1*	3	56.11	199.16	50.83–63.40	192.97–200.45	4.57	5.06	0.27	AJ517001	12.80
	*qSC-1-2*	4	60.66	229.33	57.50–63.48	208.9–240.7	3.02	3.65	0.23	AJ517001	
	*qSC-1-3*	8	14.45	7.17	9.87–22.35	5.86–16.43	5.17	6.38	−0.30	AJ517002	
SC2	*qSC-2-1*	1	41.95	61.67	40.66–45.61	60.19–83.65	3.34	3.83	0.41	AJ517003	31.40
	*qSC-2-2*	4	27.97	147.20	18.96–31.73	18.47–156.85	8.81	10.58	0.68	AJ517003	
	*qSC-2-3*	5	89.14	197.27	84.56–92.14	183.23–197.27	12.22	16.12	0.84	AJ517003	
	*qSC-2-4*	8	8.76	26.15	5.36–10.60	20.78–65.48	4.98	5.79	0.50	AJ517003	
SC3	*qSC-3-1*	3	25.61	136.60	22.24–29.26	45.76–157.17	4.27	8.03	−0.40	KB519002	41.57
	*qSC-3-2*	7	29.11	143.87	26.37–35.11	138.27–143.87	7.54	16.18	−0.57	KB519002	
	*qSC-3-3*	9	8.09	35.22	4.85–17.09	13.71–35.22	6.33	13.32	−0.52	KB519002	
SC4	*qSC-4-1*	1	32.30	44.52	29.38–35.82	39.4–62.75	6.08	8.43	0.54	KB519003	18.73
	*qSC-4-2*	1	93.48	282.61	89.95–107.15	279.59–302.14	5.33	8.36	−0.54	KB519004	
	*qSC-4-3*	5	39.47	66.02	30.35–44.91	18.85–168.63	3.81	5.32	0.43	KB519003	

a Chromosome;

b Genetic position in centimorgans (cM) of QTL with the highest LOD;

c Physical position of QTL based on the B73 reference sequence (V4);

d Genetic position range in centimorgans (cM) of QTL with the highest LOD;

e Physical position range of QTL based on the B73 reference sequence (V4);

f Percentage of the phenotypic variation explained by the additive effect of QTL;

g Additive effect of QTL;

h Parental source of QTL;

i Percentage of the phenotypic variation explained by the additive effect of all QTL.

In SC1, three QTLs (*qSC-1-1, qSC-1-2, and qSC-1-3*) were detected on chromosomes 3, 4, and 8, respectively. The phenotypic variation explained by these QTLs was 3.65–6.38% and explained by the additive effect of all QTLs was 12.8%. The parental AJ517001 allele at *qSC-1-1* and *qSC-1-2* increased the SC, while the parental AJ517002 allele increased the SC at *qSC-1-3*.

In SC2, a total of four QTLs (*qSC-2-1, qSC-2-2, qSC-2-3*, and *qSC-2-4*) were identified on chromosomes 1, 4, 5, and 8, respectively. The phenotypic variation of *qSC-2-1* and *qSC-2-4* was <10%, whereas the phenotypic variation of *qSC-2-2* and *qSC-2-3* was 10.58 and 16.12%, respectively, suggesting that *qSC-2-2* and *qSC-2-3* are two major QTLs controlling SC in SC2. The phenotypic variation explained by the additive effect of all QTLs was 31.4% and the parental AJ517003 allele at all QTLs in this population increased the SC.

In SC3, a total of three QTLs (*qSC-3-1, qSC-3-2*, and *qSC-3-3*) were detected on chromosomes 3, 7, and 9, respectively. The phenotypic variation explained by each individual QTL was 8.03-16.18%. *qSC-3-2* and *qSC-3-3* were the major QTLs explaining the phenotypic variation of 16.18 and 13.32%, respectively. The phenotypic variation explained by the additive effect of all QTLs was 41.57%, and the allele from parental KB519002 at these QTLs increased the SC.

In SC4, a total of three QTLs (*qSC-4-1, qSC-4-2, and qSC-4-3*) were identified. Among those, *qSC-4-1* and *qSC-4-2* were located on chromosome 1, and *qSC-4-3* was located on chromosome 5. The phenotypic variation explained by these QTLs was 5.32–8.43%, and the phenotypic variation explained by the additive effect of all QTLs was 18.73%. Parental KB519003 allele at *qSC-4-1* and *qSC-4-3* increased the SC, while parental KB519004 increased the SC at *qSC-4-2*.

Additionally, the additive × additive epistatic interaction analysis was also investigated. However, no epistatic interactions could be detected, indicating that the genetic component of SC in these DH populations is mainly characterized by additive gene actions.

### Genetic overlap of QTLs in the four DH populations with other populations

To evaluate genetic overlaps among different mapping populations, the 1.5-LOD support interval of QTLs in the four DH populations and in other populations for the SC previously reported were compared. QTLs with overlapping support intervals were considered common QTLs. Among the four DH populations, a 2.56 Mb overlap was observed between *qSC-2-1* and *qSC-4-2*.

**Figure 3:**
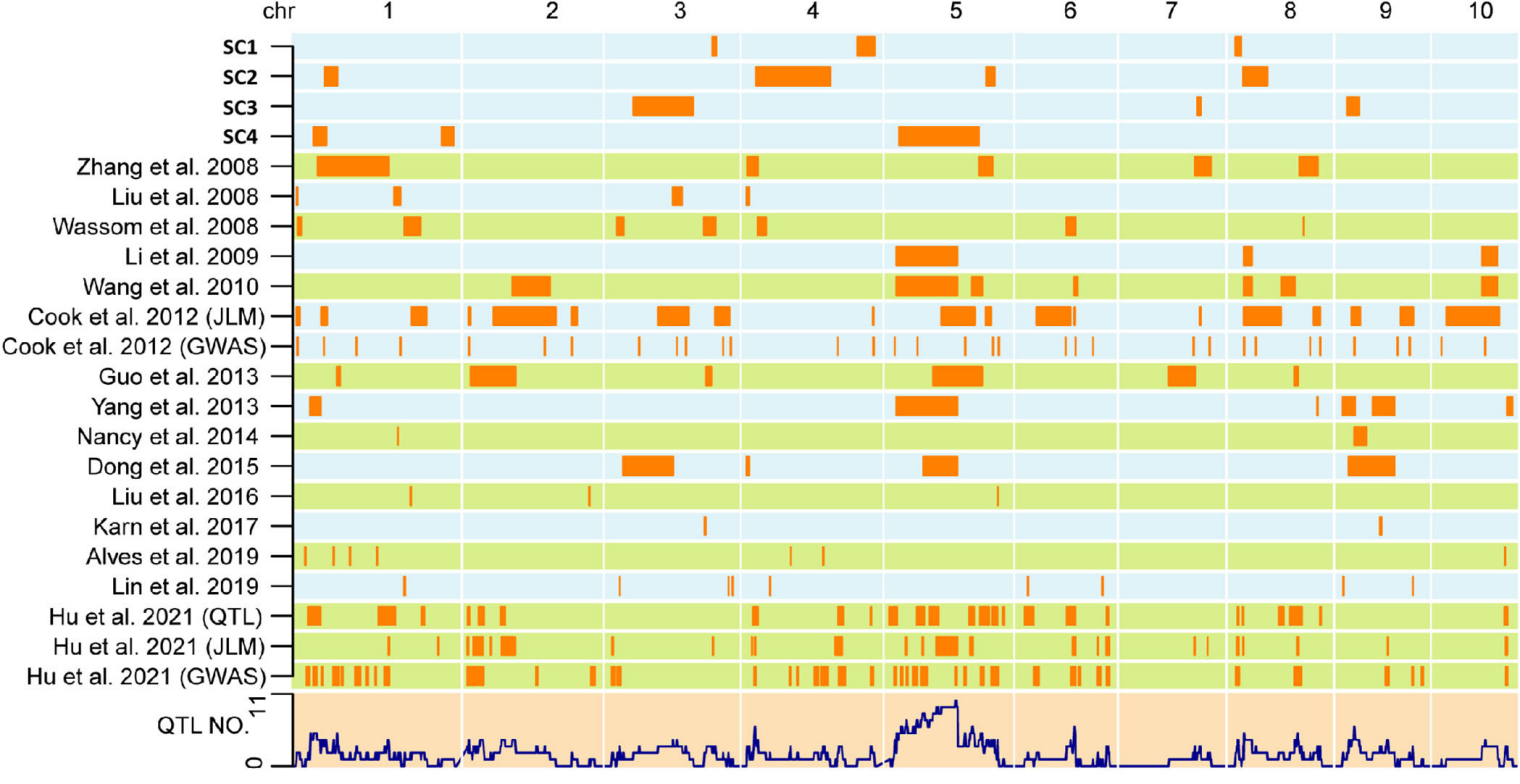
Co-localization of SC QTLs in maize kernels identified in the current and previous studies. The QTLs identified in the four DH populations are represented on top. QTLs detected in previous studies are displayed in the form of references. The lower layer shows the number of detected QTLs.

Importantly, a total of 93 overlaps were detected after comparing the four DH populations with results from all types of other populations, including RILs (Cook et al., 2012; Guo et al., 2013; Yang et al., 2013; Nancy et al., 2014; Dong et al., 2015; Lin et al., 2019; Hu et al., 2021), F_2:3_ families (Liu et al., 2008; Zhang et al., 2008; Li et al., 2009; Wang Y. Z. et al., 2010), and backcross-derived lines (Wassom et al., 2008) (Figure 3, Supplementary Table S2). For instance, *qSC-1-1* colocalized with *JLM10* (Hu et al., 2021); *qSC-1-2* colocalized with *qSTA4-3* (Hu et al., 2021); *qSC-1-3* colocalized with *JLM24* and *qSTA8-1* (Hu et al., 2021); *qSC-3-1* colocalized with *qCT-3-1* (Dong et al., 2015); *qSC-4-1* colocalized with *qSTA1-1* (Hu et al., 2021) and *qzSTA1-1-1* (Yang et al., 2013); and *qSC-4-3* colocalized with *qCT-5-1* (Dong et al., 2015), *stc5* (Guo et al., 2013), *qSTA5-3* and *qSTA5-5* (Hu et al., 2021), *qSTA1-5-1* and *qSTA1-5-2* (Wang Y. Z. et al., 2010), and *qxSTA2-5-1* (Yang et al., 2013). These results suggest that, although unique QTLs are constantly specialized in each individual population, some genetic loci may have a common effect on SC among different populations.

## Discussion

### QTL mapping precision

The starch content in maize kernel is a complex quantitative trait. Elucidation of QTLs or genes controlling phenotypic variation of SC could decipher the genetic architecture of starch in maize kernel (Huang et al., 2021). Molecular markers are useful tools to efficiently facilitate the selection process. SNP markers are the most frequent variations in genomes, and the application of SNP markers in plant breeding has guaranteed the precision of QTL mapping and genetic analysis (Bhattramakki et al., 2002; Mammadov et al., 2012; Flutre et al., 2022; Kaur et al., 2022). In the present study, a total of 13 QTLs were distributed on chromosomes 1, 3, 4, 5, 7, 8, and 9. Three QTLs (*qSC-2-2, qSC-3-1*, and *qSC-4-3*) spanned a large physical interval (111.41-149.78 Mb), partly due to the limited size of the mapping DH population. The other QTLs spanned physical intervals of <50 Mb, and two spanned <10 Mb.

### Genetic basis of SC in our DH populations

The starch content in the four DH populations examined in this study exhibited a broad range of phenotypic variations with normal distribution. The genetic analysis indicated that SC is highly heritable and the heritability is fairly high in all populations, indicating a superior genetic effect on SC in DH populations. In addition, except for environmental variation, none of the QTLs were shared by all DH populations, reflecting the complexity of SC regulation in diverse maize populations. Most of these QTLs had moderate additive effects. The PVE for each QTL ranged from 3.65 (*qSC-1-2* in SC1) to 16.18% (*qSC-3-2* in SC3). These results suggested that a few large-effect QTLs, together with a large number of minor-effect QTLs, mainly contributed to the genetic component of SC. This is consistent with the quantitative nature of SC, reflecting the complexity of starch biosynthesis and accumulation in maize kernels (Glowinski and Flint-Garcia, 2018).

The results of QTL detection derived from different studies may exhibit consistency to a certain degree across different germplasms/genetic backgrounds and environments. Indeed, QTLs including *qSC-2-2, qSC-2-3, qSC-3-2*, and *qSC-3-3* with the highest contribution to phenotypic variation (10.58–16.18%) displayed a high degree of overlap with regions associated for SC in multiple former studies (Wassom et al., 2008; Zhang et al., 2008; Cook et al., 2012; Yang et al., 2013; Nancy et al., 2014; Dong et al., 2015; Alves et al., 2019; Lin et al., 2019; Hu et al., 2021). Meanwhile, our QTLs with the second largest effect (3.65–8.43%) showed less degree of overlap with other studies (Liu et al., 2008; Li et al., 2009; Wang Y. Z. et al., 2010; Guo et al., 2013). Moreover, *qSC-4-2* is a new QTL, which is definitely worth conducting further research on this QTL *via* near-isogenic lines (NILs), fine mapping, molecular marker-assisted selection (MAS), and ultimate cloning. Furthermore, considering that each of the four DH populations was developed from biparental crosses, the derived QTLs could only explain the variation between the two parents in regulating SC. Therefore, it would be necessary to assess SC in extra germplasms to get a comprehensive insight into the genetic factors regulating the natural variation of SC.

### Importance of QTLs relevant to SC in maize genetic and breeding

**Figure 4:**
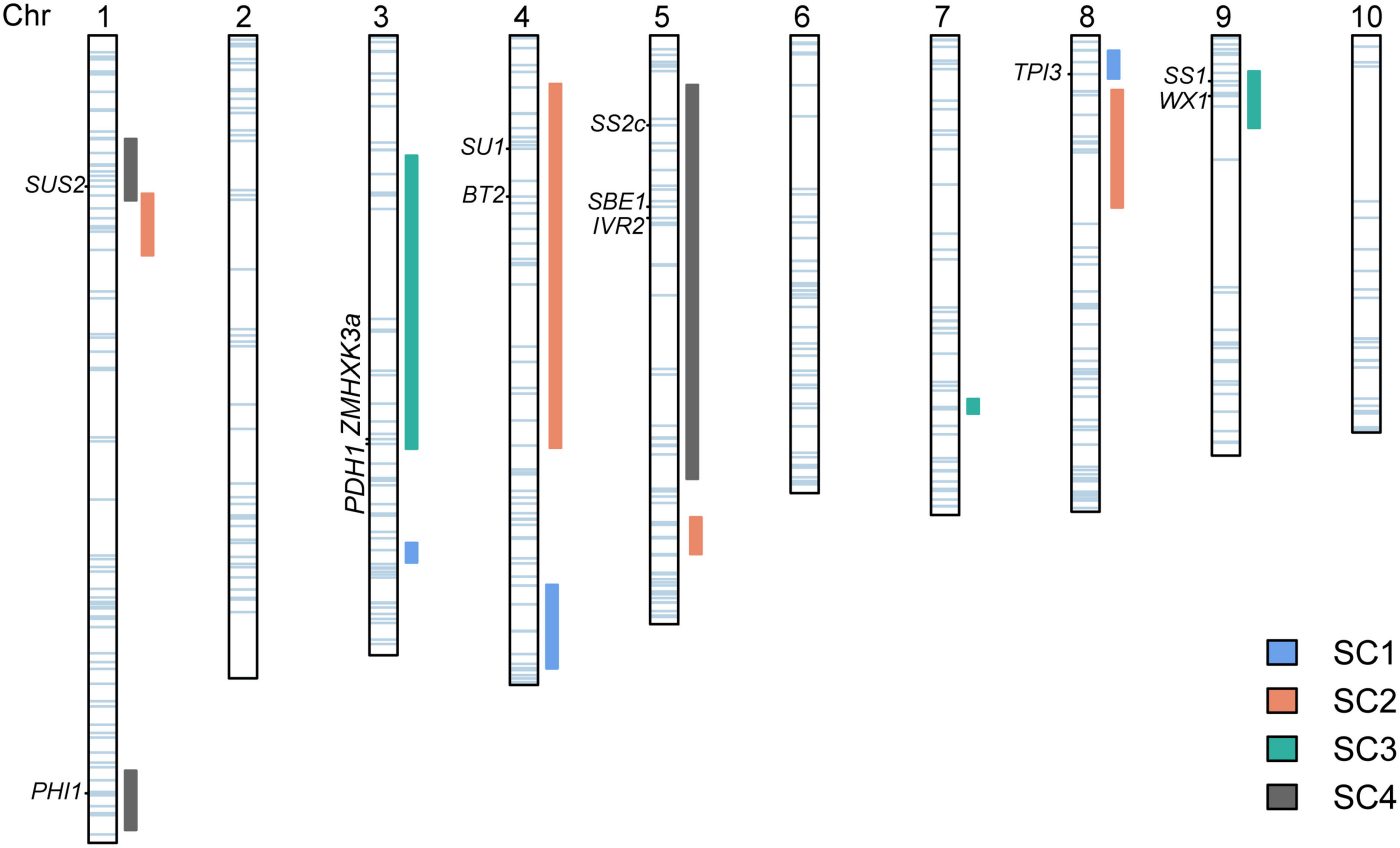
Association of candidate genes with kernel starch QTLs. The QTLs identified in four DH populations are represented as vertical rectangles of different colors next to each chromosome. The horizontal light blue bars on each chromosome show the positions of the 471 identified genes. The left labels denote known genes that co-localized with the QTLs. The vertical dark green lines indicate the positions of 12 well-known genes encoding key enzymes in maize starch metabolism.

The starch content in maize kernel is regulated by many genes (Zhong et al., 2020). Outstanding progress has been made in the understanding of the genetic and biochemistry of starch synthesis, which involves the coordinated activities of a series of starch metabolic enzymes, including sucrose synthase (SUS), adenosine 5'diphosphate-glucose pyrophosphorylase (AGPase), starch synthases (SSs), starch branching enzymes (BEs), and starch debranching enzymes (DBEs) (Nelson and Pan, 1995). Candidate genes underlying QTLs associated with SC may be suggested in view of the co-location of QTLs with genes encoding these enzymes (Prioul et al., 1997; Thévenot et al., 2005). Moreover, the co-location analysis could provide information about the functional relationships between gene expression and some QTLs of the starch biosynthesis pathway (Thévenot et al., 2005). Our study involved 471 genes in the glycolysis, sucrose, and starch pathway, of which 123, including 12 well-known genes encoding key enzymes in maize starch regulation of synthesis and metabolism, were located within QTL intervals (Figure 4, Supplementary Table S3).

The starch synthesis pathway in maize endosperm initiates with the cleavage of sucrose into fructose and UDP-glucose, which is catalyzed by SUS (Nelson and Pan, 1995). *Sus2* is one of three SUS-encoding genes in maize (Duncan et al., 2006) and is located inside *qSC-4-1*. Transactivation of *Sus1* and *Sus2* by Opaque2 is an essential supplement to sucrose synthase-mediated endosperm filling in maize, indicating that *Sus2* might have a unique role in cytoplasmic sucrose metabolism (Deng et al., 2020). *IVR2* in *qSC-4-3* is annotated to be one of the vacuolar invertases in the maize genome (Kim et al., 2000). It is also a key enzyme of carbon metabolism in both source and sink tissues that irreversibly hydrolyze sucrose to fructose and glucose and regulates sugar accumulation in sink organs (Juárez-Colunga et al., 2018). This gene was also identified in a GWAS study and linked with natural variation in the amylose content (Li et al., 2018). *Brittle2* (*Bt2*) in *qSC-2-1* encodes the small unit of AGPase, which catalyzes the upstream products converting into adenosine 5'diphosphate-glucose (ADPG), that is, the glucosyl donors of amylose and amylopectin (Preiss et al., 1990) and the first rate-limiting enzyme in the starch biosynthetic pathways (Ma et al., 2021; Finegan et al., 2022). *ZMHXK3a* in *qSC-3-1, PHI1* in *qSC-4-2*, and *TPI3* in *qSC-1-3* also play very important roles in glycolysis and starch metabolism. *ZMHXK3a* encodes a hexokinase, which catalyzes the conversion of glucose to glucose-6-phosphate (Xiao et al., 2016). *PHI1* encodes phosphohexose isomerase 1 that converts fructose into glucose-1-phosphate and *TPI3* encodes triosephosphate isomerase isozymes representing the cytosol and involved in glycolysis (Wendel et al., 1989). *WX1* and *SS1* in *qSC-3-3* and *SS2C* in *qSC-4-3* belong to starch synthases. *WX1* encoding granule-bound starch synthase I (GBSSI) is solely responsible for amylose production (Shure et al., 1983), whereas *SS1* (Knight et al., 1998) and *SS2C* (Yan et al., 2009) encoding soluble starch synthases are responsible for the synthesis of amylopectin. *SBE1* in *qSC-4-3* encodes an isozyme of SBE that generates amylopectin by cleaving internal amylase a-(1,4) glucosidic bonds and transferring the reducing ends to C6 hydroxyls to form a-(1,6) bonds (Jiang et al., 2013). Besides, *SBE1* was shown to be related to amylose content and starch molecular structure (Zhong et al., 2021). *SU1* in *qSC-2-2* encodes the DBE isoforms ISA1, which is responsible for the ordering of branch linkages and linear chains as depicted in the widely accepted cluster model of the amylopectin structure (James et al., 1995).

## Conclusion

In the present study, we interpreted the genetic basis and QTL mapping of SC in four DH populations. A total of 13 QTLs accounted for 12.80–41.57% of the starch variation, with four QTLs explaining that more than 10% of the phenotypic variation were identified. One novel QTL has never been reported in any previous studies. These results indicated that the phenotypic variation in SC is coordinated by large-effect QTLs and minor-effect QTLs. Our results further enhanced the understanding of genetic variation in SC and offered prospective routes to modify SC through molecular marker-assisted selection in the maize breeding program.

## Data availability statement

The original contributions presented in the study are publicly available. This data can be found here: https://doi.org/10.6084/m9.figshare.20188547.v2.

## Author contributions

RZ, CZ, and MW conceived and designed the experiments. XZ, CD, and HG performed the research. MW analyzed the data. XZ and MW wrote the manuscript. All authors read and approved the manuscript, contributed to the article, and approved the submitted version.

## Funding

This research was funded by the National Natural Science Foundation of China, grant number 32201798.

## Conflict of interest

Author CZ was employed by Maize Yufeng Biotechnology LLC (Beijing, China). The remaining authors declare that the research was conducted in the absence of any commercial or financial relationships that could be construed as a potential conflict of interest.

## Publisher's note

All claims expressed in this article are solely those of the authors and do not necessarily represent those of their affiliated organizations, or those of the publisher, the editors and the reviewers. Any product that may be evaluated in this article, or claim that may be made by its manufacturer, is not guaranteed or endorsed by the publisher.
