# Supplementary material for: Genetic dissection of QTLs for starch content in four maize DH populations
Source: Front Plant Sci. 2022 Oct 6;13:950664. doi: 10.3389/fpls.2022.950664 (PMC9583244; doi:10.3389/fpls.2022.950664)
Supplement: Supplementary file 1 [file Presentation_1.pdf]

Supplementary Material

Supplementary Figures

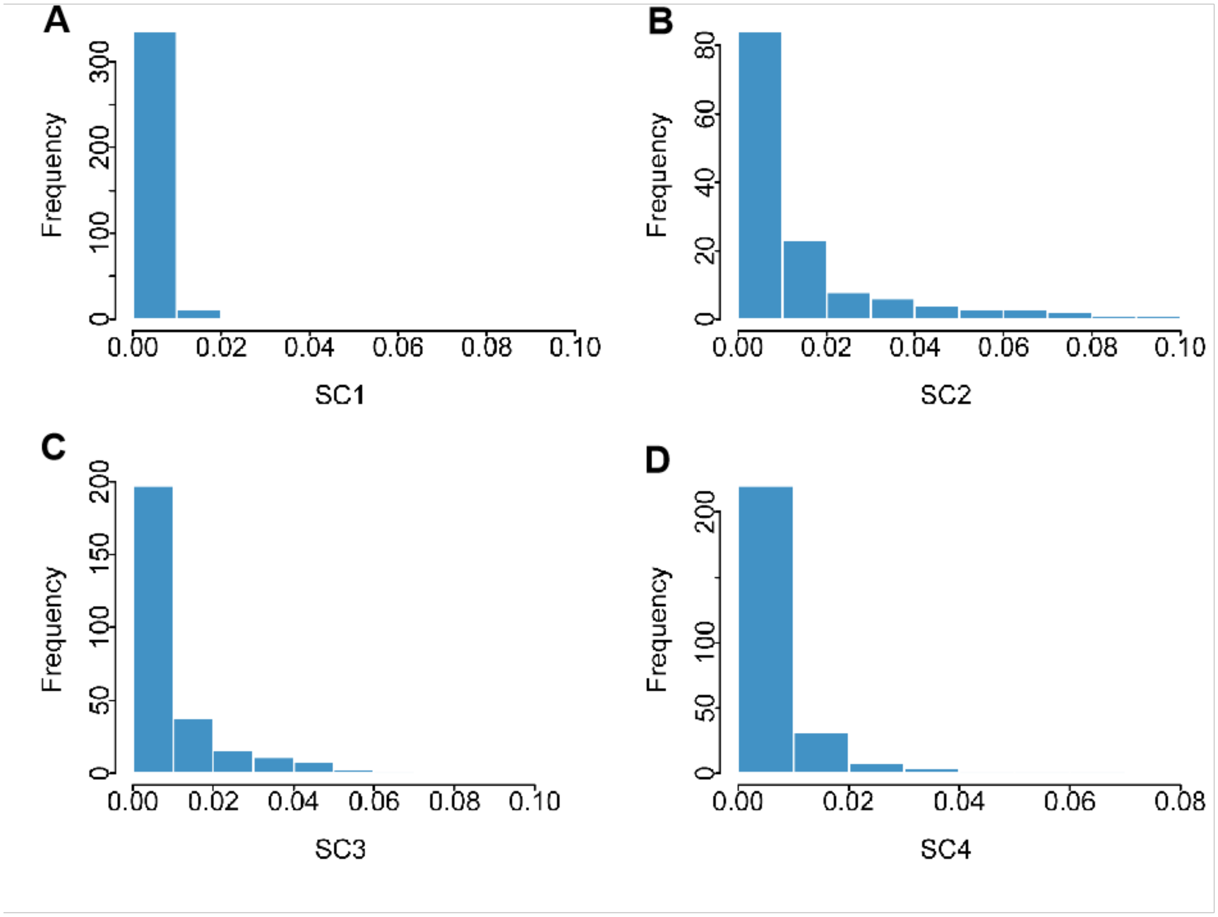

Supplementary Figure 1. Distribution of the missing rate for SNP in each line. (A), (B), (C) and (D) designated SC1, SC2, SC3 and SC4, respectively.

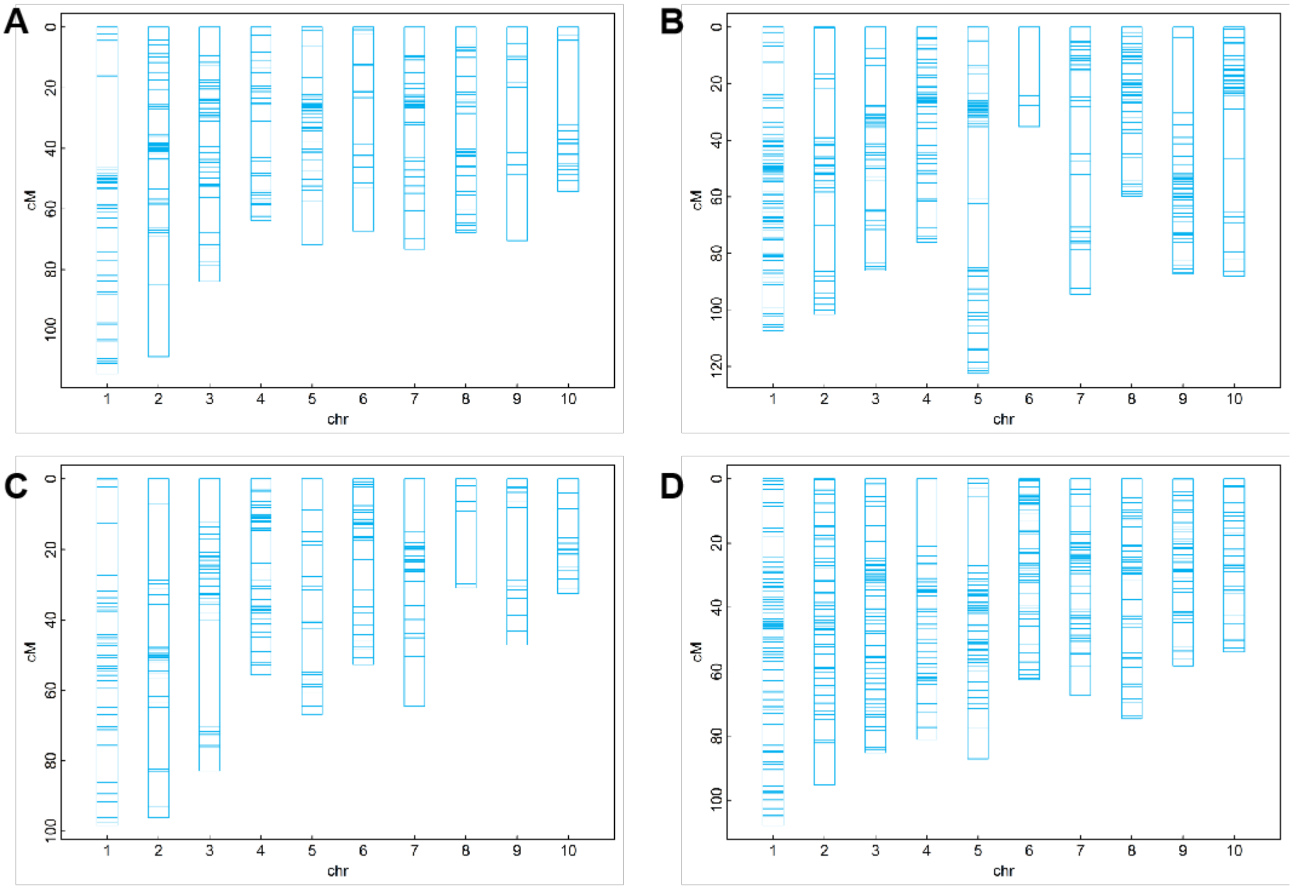

Supplementary Figure 2. Linkage maps of four DH populations. The horizontal light blue bars on each chromosome showed the genetic position of each SNP. (A), (B), (C) and (D) designated SC1, SC2, SC3 and SC4, respectively.
